# Supplementary material for: Molecular Events behind the Selectivity and Inactivation Properties of Model NaK-Derived Ion Channels
Source: Int J Mol Sci. 2022 Aug 17;23(16):9246. doi: 10.3390/ijms23169246 (PMC9409022; doi:10.3390/ijms23169246)
Supplement: Supplementary file 1 [file ijms-23-09246-s001.zip › ijms-1831969-supplementary.pdf]

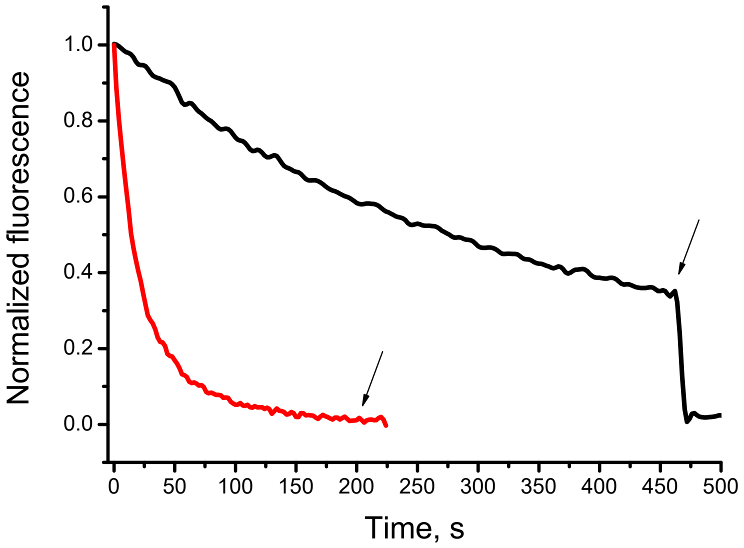

Figure S1. Normalized K^+ flux of NaK (black) and NaK2K (red) channels in asolectin liposomes measured through the ACMA/CCCP fluorescence assay. The addition of CCP sets the time zero of the experiment. Valinomycin was added at the end of the recording (black arrow) to induce channel-independent K^+ efflux.

Table S1. Analysis of the fluorescence intensity decays obtained at room temperature for the NaK and NaK2K channels in 20 mM Hepes, 5 mM DDM, 5 mM NMG, pH 7 buffers (λ_{ex}= 300 nm; λ_{em}= 345 nm).

Channel	Salt	α_1	τ_1 (ns)	α_2	τ_2 (ns)	α_3	τ_3 (ns)	$<\tau>_1$ (ns)	$<\tau>_2$ (ns)
NaK	Na^{+*}	0.41 [0.18-0.64]	0.69 [0.45-0.92]	0.29 [0.15-0.42]	3.0 [1.9-4.1]	0.30 [0.18-0.42]	6.7 [5.8-7.5]	3.1 [2.8-3.5]	5.1 [4.9-5.4]
	K^{+*}	0.34 [0.20-0.47]	0.64 [0.55-0.73]	0.23 [0,19-0.27]	2.4 [1.8-2.9]	0.43 [0.32-0.55]	6.1 [5.9-6.3]	3.4 [2.8-4.0]	5.1 [4.9-5.3]
NaK2K	Na^{+**}	0.669 [0.59-0.75]	0.72 [0.70-0.75]	0.20 [0.15-0.25]	2.7 [2.4-3.0]	0.13 [0.09-0.17]	7.3 [6.7-8-0]	2.0 [1.7-2.3]	4.4 [3.9-4.9]
	K^{+*}	0.36 [0.20-0.52]	0.61 [0.46-0.75]	0.21 [0.20-0.23]	2.4 [1.7-3.1]	0.4 [0.3-0.6]	6.1 [5.5-6.6]	3.3 [2.6-3.9]	5.1 [4.8-5.3]

α_i and τ_i are the normalized amplitude and the lifetime of the i th decay component. The amplitude-weighted average fluorescence lifetime, $<\tau>_1$ and the average lifetime $<\tau>_2$, was calculated as in [1]. *The concentration of salt used was 100 mM, **The concentration of NaCl used was 200 mM, The calculated $<\tau>_1$ and $<\tau>_2$ represent the mean of at least three independent experiments. Values in brackets correspond to the 95% confidence intervals.

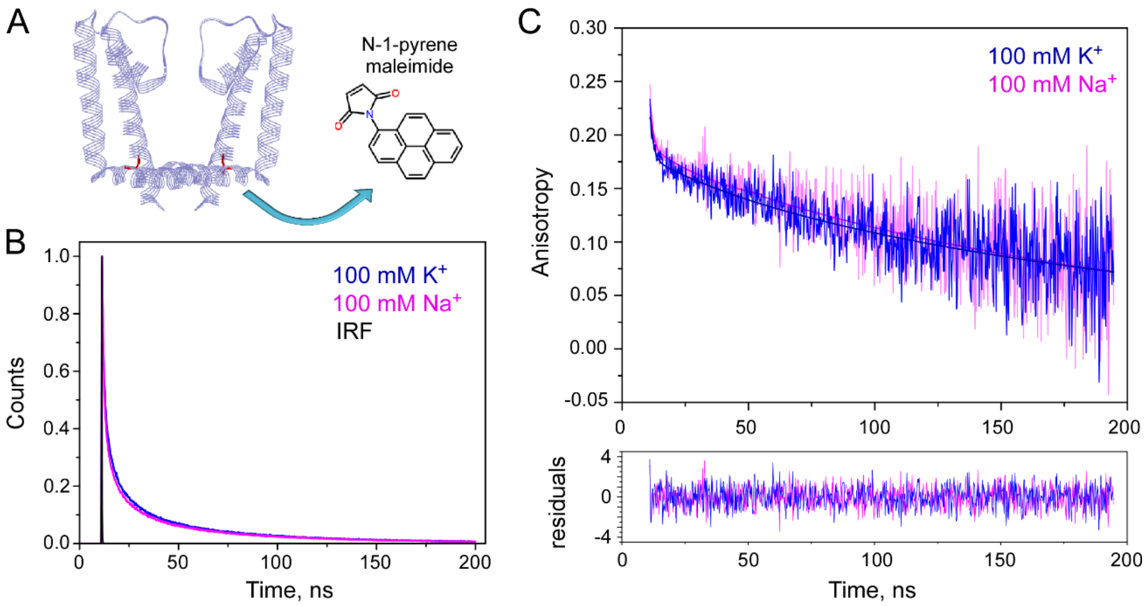

Figure S2. Determination of the global rotational time of the NaK-DDM complex in 100 mM K^+ (blue lines) and 100 mM Na+ (magenta lines). **Panel A**: schematic representation (side view) of two of the four monomers of the full-length wild-type NaK channel (PDB: 2AHZ) and the location of the native C15 position (red sticks). These residues were covalently attached to a N-1-pyrene dye following the maleimide chemistry. **Panels B and C** illustrate representative time-resolved fluorescence and anisotropy decays, respectively (λ_{ex}= 335 nm; λ_{em}= 400 nm). No significant changes were observed when K^+ or Na^+ were present in the buffer (20 mM Hepes, 1 mM DDM, pH 7.0). The solid lines in panel C represent the best fit of Eq. 3 to the anisotropy decays. The intensity-weighted average lifetime was ~ 50 ns and the global rotational correlation time, ϕ_g, 40 ± 3 ns.

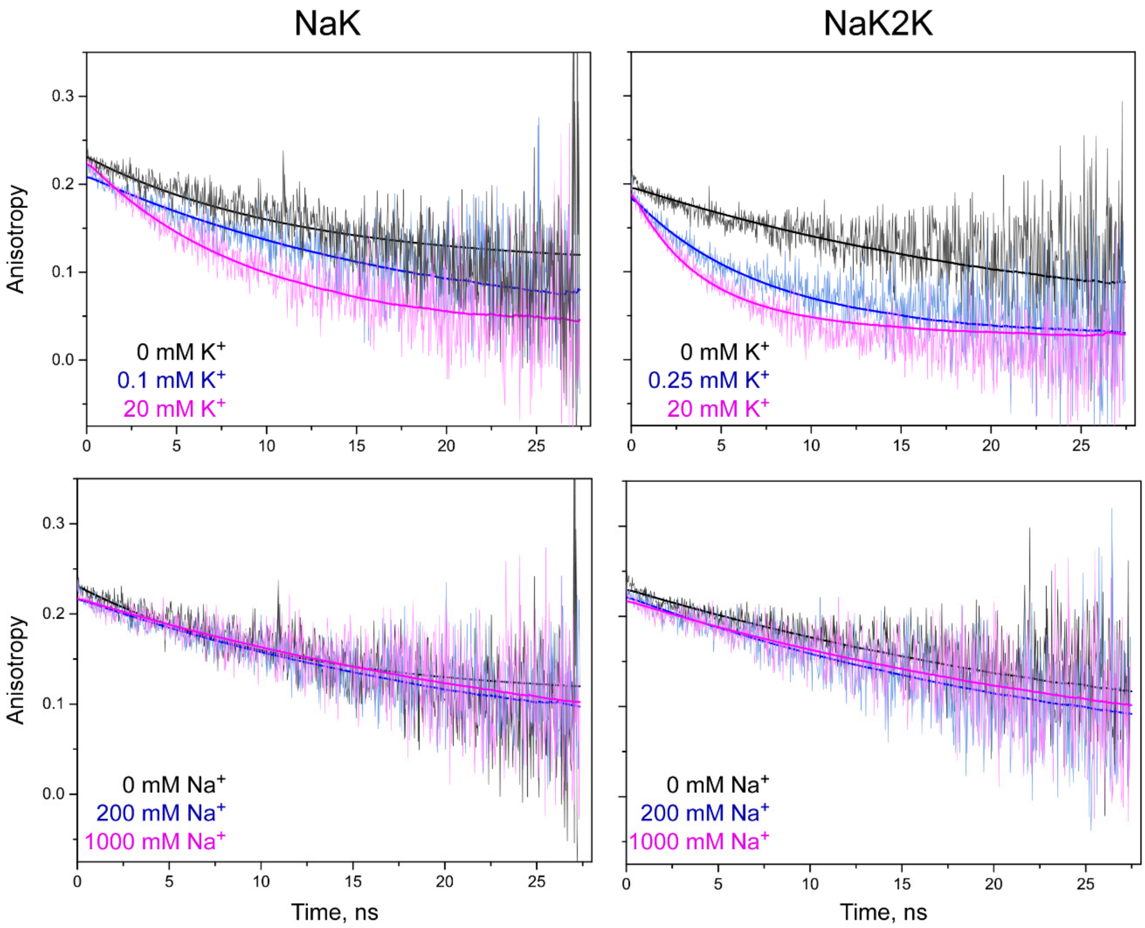

Figure S3. Representative time-resolved anisotropy decays of NaK (left column) and NaK2K (right column) channels in the presence of increasing amount of K^+ or Na^+. The straight lines correspond to the best fit of Equation (3) to the data.

References

1. Renart, M.L.; Giudici, A.M.; Poveda, J.A.; Fedorov, A.; Berberan-Santos, M.N.; Prieto, M.; Díaz-García, C.; González-Ros, J.M.; Coutinho, A. Conformational Plasticity in the KcsA Potassium Channel Pore Helix Revealed by Homo-FRET Studies. *Sci. Rep.* **2019**, *9*, 6215–6228. https://doi.org/10.1038/s41598-019-42405-5.
